# Association between serum LDL‐C concentrations and risk of diabetes: A prospective cohort study

**DOI:** 10.1111/1753-0407.13440

**Published:** 2023-07-17

**Authors:** Jiaojiao Huang, Hong Lin, Shuangyuan Wang, Mian Li, Tiange Wang, Zhiyun Zhao, Yu Xu, Min Xu, Jieli Lu, Yuhong Chen, Guang Ning, Weiqing Wang, Yufang Bi, Long Wang

**Affiliations:** ^1^ Department of Endocrine and Metabolic Diseases Shanghai Institute of Endocrine and Metabolic Diseases, Ruijin Hospital, Shanghai Jiao Tong University School of Medicine Shanghai China; ^2^ Key Laboratory for Endocrine and Metabolic Diseases of the National Health Commission Shanghai National Clinical Research Center for Metabolic Diseases, Shanghai National Center for Translational Medicine, Ruijin Hospital, Shanghai Jiao Tong University School of Medicine Shanghai China

**Keywords:** diabetes mellitus, LDL‐C, prospective, risk, LDL‐C, 糖尿病, 前瞻性, 风险

## Abstract

**Background:**

Low‐density lipoprotein cholesterol (LDL‐C) and diabetes mellitus are both modifiable risk factors for cardiovascular disease; however, whether elevated LDL‐C levels confer a risk for diabetes remains unclear.

**Objective:**

We aimed to examine the association between serum LDL‐C concentrations at baseline and the risk of developing diabetes at follow‐up in the general population of Chinese adults.

**Methods:**

This study included 5274 adults aged ≥ 40 years from a community cohort who were without diabetes and followed for a median of 4.4 years. A standard 75‐g oral glucose tolerance test was conducted at baseline and follow‐up visits to diagnose diabetes. Logistic regression models and a restricted cubic spline were used to examine the association between baseline serum LDL‐C levels and the risk of diabetes development. Subgroup analyses were conducted stratifying on age, sex, body mass index, hypertension, family history of diabetes, and LDL‐C levels.

**Results:**

A total of 652 participants (12%) developed diabetes during the follow‐up period. Compared to quartile 1 of serum LDL‐C, quartiles 2, 3, and 4 were associated with a 30%, 33%, and 30% significantly higher risk of diabetes, respectively after adjustment for confounders including homeostatic model assessment for insulin resistance. The linear relationship between baseline LDL‐C down to 30.1 mg/dL and incident diabetes was demonstrated by restricted cubic spline analysis, and each 1‐SD increase in LDL‐C concentration (28.5 mg/dL) was associated with a 12% increase in the risk of diabetes (odds ratio 1.12, 95% confidence interval 1.03–1.22).

**Conclusion:**

In this community‐based general population, higher serum LDL‐C levels were linearly associated with an elevated risk of incident diabetes.

## Graphical abstract

**Highlights**

- Serum low‐density lipoprotein cholesterol (LDL‐C) levels were positively and significantly associated with a high risk of incident diabetes.
- Even in participants with normal LDL‐C levels (<130 mg/dL), a 1‐SD increase in LDL‐C levels was significantly associated with a higher risk of diabetes development.
- More attention and health management should be implemented on patients with elevated LDL‐C to reduce diabetes risk.

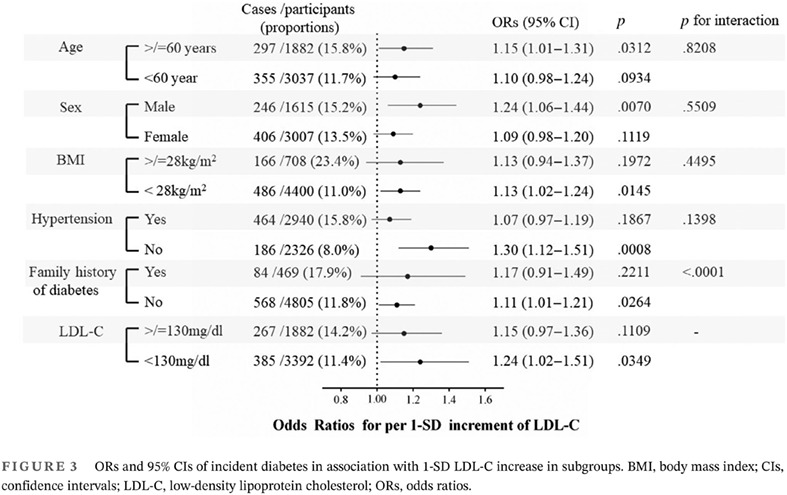

## 摘要

背景: 低密度脂蛋白胆固醇(LDL‐C)和糖尿病都是心血管疾病的可改变风险因素; 然而，升高LDL‐C水平是否会带来糖尿病的风险仍不清楚。本文旨在探讨在中国成年人中的基线血清LDL‐C浓度与随访期间糖尿病风险之间的关联。

方法: 本研究包括5274名基线年龄≥40岁的社区队列参与者，他们无糖尿病且中位随访时间为4.4年。在基线和随访期间进行75g口服糖耐量试验以诊断糖尿病。使用逻辑回归模型和限制性立方样条来探讨基线血清LDL‐C水平与糖尿病风险之间的关联。根据年龄、性别、BMI、高血压、糖尿病家族史和LDL‐C水平进行亚组分析。

结果: 在随访期间，共有652名(12%)参与者发生糖尿病。校正包括HOMA‐IR在内的混杂因素后，与血清LDL‐C四分位组1相比，四分位组2、3和4的糖尿病风险分别显著增加30%、33%和30%。限制性立方样条分析显示基线LDL‐C降至30.1mg/dl与糖尿病发病之间存在线性关系，LDL‐C浓度每增加1个标准差(28.5mg/dl)，糖尿病发病风险增加12% (OR 1.12, 95%CI 1.03 ~ 1.22)。

结论: 在基于社区的一般人群中，血清LDL‐C水平升高与新发糖尿病的风险增加呈线性关系。

## 1 INTRODUCTION

With an estimated 537 million adults living with diabetes globally in 2021, the prevalence of diabetes is a major health problem worldwide and is still on the rise.^1^ China has the world's largest number of patients with diabetes, which is reported to account for 12.4% of the total adult Chinese population.^2^ Almost one in two adults living with type 2 diabetes mellitus is undiagnosed^1^; therefore, identifying the high‐risk population for early prevention is of great importance to slowing down the rising trend of diabetes.

Diabetic dyslipidemia is a common condition in patients with diabetes, and both hypercholesterolemia and diabetes predispose individuals to cardiovascular diseases (CVD). The relationship between serum cholesterol levels and risks of diabetes has been investigated but findings were inconsistent. Early clinical trials have reported that statin therapy, a classical low‐density lipoprotein cholesterol (LDL‐C) lowering treatment that inhibits endogenous cholesterol synthesis, is associated with a slightly increased risk of diabetes development, which might be partly explained by its pharmacological mechanism.^3^ Additionally, researchers have reported that exceptionally low LDL‐C concentrations occurring in the absence of statin treatment are significantly associated with type 2 diabetes risk.^4^ Notably, serum LDL‐C levels in the normal range have also been found to be inversely associated with the incidence risk of diabetes in both epidemiological studies and Mendelian randomization analysis.^5^ Thus far, it seems that lower levels of serum LDL‐C are associated with a greater risk of diabetes. Intriguingly, data published in 2018 from a small sample of the Iranian population of diabetes‐related families presented a positive association between serum LDL‐C levels and the risk of new‐onset diabetes during a mean follow‐up of 10 years. Later, Mendelian randomization analyses using 23 single nucleus polymorphisms that were significantly related to LDL‐C (*p* ≤ 5.0 × 10^−8^) identified by the Global Lipids Genetics Consortium from 188 577 individuals of European ancestry reported a causal association between elevated serum LDL‐C levels and increased diabetes risks.^6^

Therefore, genetic as well as epidemiological analyses have shown contradictory findings on the association between circulating LDL‐C levels and diabetes risk. Furthermore, a cross‐sectional analysis of 9892 Chinese participants with hypertension showed a U‐shaped association between LDL‐C levels and diabetes.^7^ However, prospective analyses on the association between serum LDL‐C concentrations and the risk of new‐onset diabetes in Chinese adults are scarce. Therefore, our study aimed to investigate the relationship between LDL‐C and diabetes risk and to identify factors that may potentially modify the association using a large community‐based prospective cohort of Chinese adults.

## 2 METHODS

### 2.1 Study population

**FIGURE 1:**
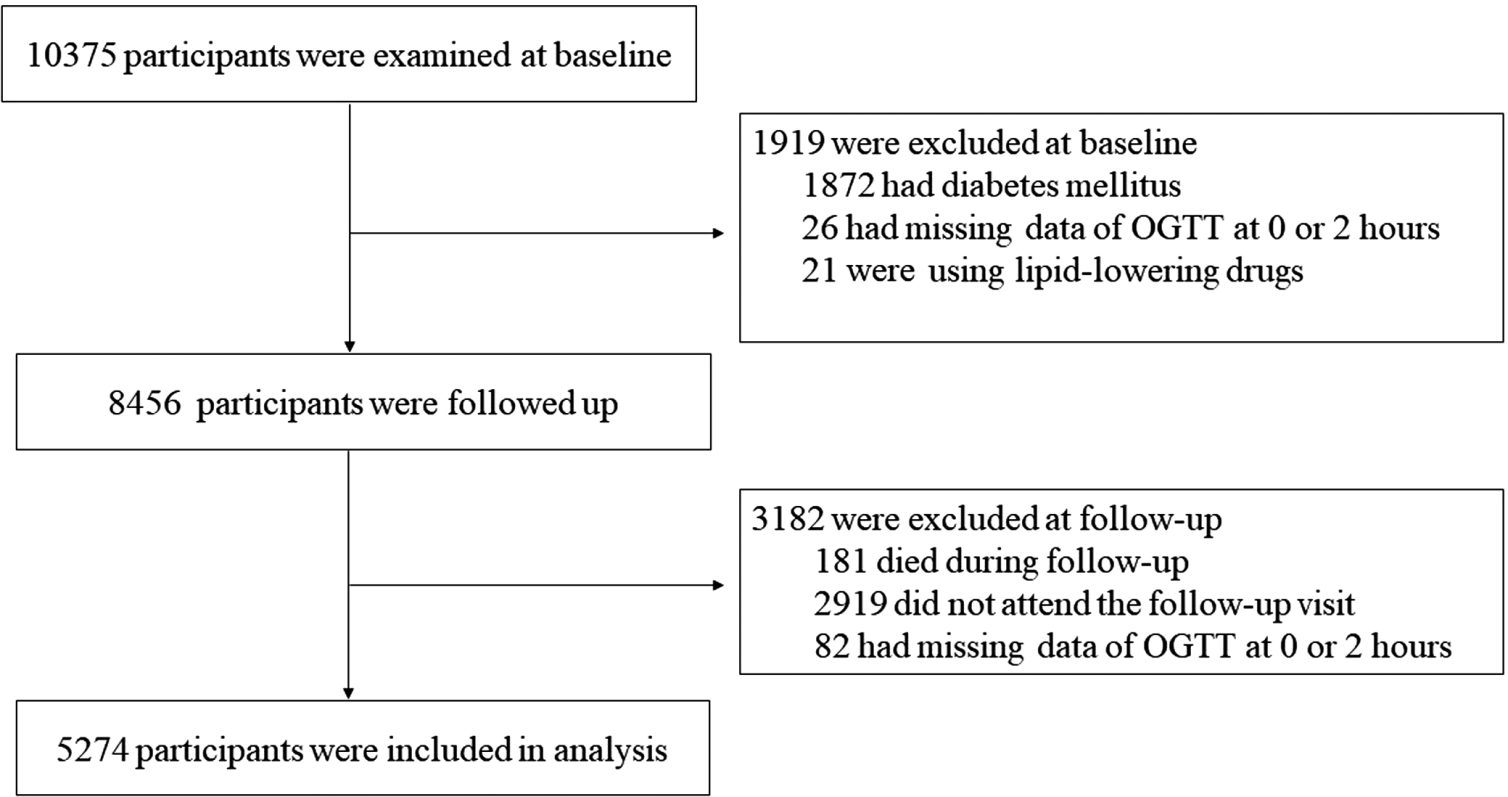
Flow chart of study population. OGTT, oral glucose tolerance test.

The participants were from an ongoing cohort study in Jiading District, Shanghai, China. The study design and detailed methods have been described previously.^8^, ^9^ Briefly, registered residents from two communities in Jiading District were invited to attend a comprehensive health examination, and 10 375 residents aged 40 years or older were recruited between March and August 2010. All the participants completed a standard questionnaire and a set of clinical measurements. A standard 75‐g oral glucose tolerance test (OGTT) was performed in participants who did not already receive glucose‐lowering treatment. Fasting and 2‐h post‐load plasma glucose levels were measured. During August 2014 and May 2015, all participants were invited to attend a follow‐up visit and received the OGTT again for a reevaluation of glucose levels. In the current study, we excluded participants who had (a) preexisting diabetes (*n* = 1872); (b) missing values of 0 or 2‐h glucose levels (*n* = 26); and (c) lipid‐lowering drugs (*n* = 21) at baseline. We further excluded participants who (a) died during follow‐up (*n* = 181); (b) did not participate in the follow‐up visit (*n* = 2919); and (c) had missing values of 0 or 2‐h glucose levels at follow‐up (*n* = 82). Details of the selection of the study participants are shown in Figure 1. Ultimately, 5274 participants were included in the current analysis. Those who were included and those who did not participate in the follow‐up visit had similar demographic and metabolic characteristics, as well as baseline glucose levels.

### 2.2 Ethics statement

The Institutional Review Board of Ruijn Hospital affiliated with Shanghai Jiao Tong University School of Medicine approved the study protocol, according to the principles of the Declaration of Helsinki. All the participants provided written informed consent before data collection.

### 2.3 Data collection

Face‐to‐face interviews were performed at the baseline and the follow‐up visit by trained study personnel using a standard questionnaire that collected information on sociodemographic characteristics, lifestyle factors, medical history, and current medication use. Information on intensity, duration, and frequency of physical activity was obtained using the short form of the International Physical Activity Questionnaire.^10^ Current smokers or drinkers were defined as those who had smoked cigarettes or consumed alcohol regularly in the past 6 months.

Anthropometric measurements, including body height and weight, were performed using calibrated instruments according to a standard protocol. Body mass index (BMI) was calculated as body weight in kilograms divided by height in meters squared. Blood pressure was measured, after at least 5 min of sitting rest, on the nondominant arm of each participant using an automated device (OMRON Model HEM‐752 FUZZY; Omron Co., Dalian, China). Three consecutive measurements with 1 min intervals were recorded. The mean of the three measurements was calculated and used for analysis.

All participants underwent a standard OGTT after an overnight fast of at least 10 h, and venous blood samples were obtained at 0 and 2 h during the test. Blood glucose levels were measured with an autoanalyzer (Modular P800; Roche, Basel, Switzerland) using the glucose oxidation method. The levels of total cholesterol, high‐density lipoprotein cholesterol (HDL‐C), LDL‐C, and triglycerides were measured with an autoanalyzer (Modular E170; Roche, Basel, Switzerland) using the chemiluminescence method.

### 2.4 Definition

According to the 1999 World Health Organization criteria, diabetes was defined as a fasting plasma glucose (FPG) level ≥ 126 mg/dL, and/or a 2‐h post‐load plasma glucose (PPG) level ≥ 200 mg/dL, and/or the use of any glucose‐lowering medications. In those without diabetes, impaired fasting glucose was defined as FPG ≥ 110 mg/dL and < 126 mg/dL and impaired glucose tolerance was defined as PPG ≥ 140 mg/dL and < 200 mg/dL. Hypertension was defined as a systolic blood pressure (BP) ≥ 140 mm Hg, and/or diastolic BP ≥ 90 mm Hg, and/or taking any BP‐lowering medications. Dyslipidemia was defined as total cholesterol ≥ 240 mg/dL, and/or LDL‐C ≥ 160 mg/dL, and/or triglycerides ≥ 200 mg/dL, and/or HDL‐C ≤ 40 mg/dL, and/or taking any lipid‐lowering medications.^11^ History of CVD was defined as a self‐reported previous diagnosis of stroke, myocardial infarction, or other coronary heart disease. A family history of diabetes was defined as the presence of at least one first‐degree family member diagnosed with diabetes. Ideal physical activity was defined as ≥ 150 min/week moderate intensity or ≥ 75 min/week vigorous intensity or ≥ 150 min/week moderate + vigorous intensity activity.^12^

### 2.5 Statistical analysis

Baseline characteristics of the study population are demonstrated according to LDL‐C levels. Continuous variables are presented as means ± SDs for normally distributed variables or as medians (interquartile ranges) for skewed variables. Categorical variables are presented as numbers and proportions.

We examined the pattern of association between LDL‐C levels and incident diabetes using restricted cubic splines with knots at the 5th, 50th, and 95th percentiles of the distribution of LDL‐C levels. The LDL‐C level was also fit as a continuous variable to estimate the risk of developing diabetes in association with a 1‐SD increase in LDL‐C levels. For all regression analyses, we used three models. Model 1 was adjusted for age and sex. Model 2 was adjusted for age, sex, educational level (≥ 9 years of education, yes or no), physical activity (ideal level, yes or no), current smoking (yes or no), current drinking (yes or no), family history of diabetes (yes or no), hypertension (yes or no), BMI, triglycerides, and HDL‐C. Model 3 was further adjusted for homeostatic model assessment for insulin resistance (HOMA‐IR) based on model 2. The association between a 1‐SD increase in LDL‐C levels and incident diabetes was examined in subgroups of age (< and ≥ 60 years), sex, BMI (< and ≥ 28 kg/m^2^), hypertension (with and without), family history of diabetes (with and without), and LDL‐C levels (< and ≥ 130 mg/dL). Several sensitivity analyses were conducted by excluding participants with baseline diseases.

All statistical analyses were performed using the SAS software (version 9.4; SAS Institute, Cary, NC, USA). A 2‐sided *p* value of less than .05 was considered statistically significant.

## 3 RESULTS

### 3.1 Basic characteristics of the study population

Study participants' characteristics are presented in Table 1 according to quartiles of serum LDL‐C concentrations at baseline. Those with higher serum LDL‐C levels were more likely to be older, female, not currently smoking or drinking, and had completed less education (all *p* <.0001). Higher serum LDL‐C levels were also associated with worse metabolic profiles, including higher BMI, BP, FPG, PPG, and HOMA‐IR (all *p* < .0001).

**TABLE 1:** Characteristics of study population at baseline, according to quartiles of serum LDL‐C concentrations.

	Quartile 1	Quartile 2	Quartile 3	Quartile 4	*p*
LDL‐C (mg/dL)	30.1–101.2	101.5–120.5	120.8–142.1	142.5–352.5	
No. of participants	1329	1295	1326	1324	
Age (years)	56.0 ± 9.3	56.7 ± 8.8	58.3 ± 8.6	58.6 ± 7.6	<.0001
Male sex, *n* (%)	576 (43.3)	509 (39.3)	418 (31.5)	358 (27.0)	<.0001
≥9 years' education, *n* (%)	926 (69.89)	848 (66.0)	841 (63.9)	791 (59.9)	<.0001
Current drinkers, *n* (%)	167 (12.9)	125 (10.0)	116 (9.0)	10.3 (8.0)	.0003
Current smokers, *n* (%)	325 (25.2)	283 (22.7)	212 (16.5)	195 (15.3)	<.0001
Ideal physical activity, *n* (%)	286 (21.5)	262 (20.2)	268 (20.2)	255 (19.3)	.5484
Family history of diabetes, *n* (%)	97 (7.3)	120 (9.3)	120 (9.1)	132 (10.0)	.0967
Body mass index (kg/m^2^)	24.4 ± 3.2	24.9 ± 3.2	25.1 ± 3.1	25.4 ± 3.0	<.0001
Systolic blood pressure (mm Hg)	136.2 ± 18.9	138.8 ± 19.4	140.9 ± 19.4	142.7 ± 19.5	<.0001
Diastolic blood pressure (mm Hg)	81.4 ± 10.2	82.3 ± 10.1	83.6 ± 10.3	83.7 ± 10.2	<.0001
Total cholesterol (mg/dL)	167.9 ± 29.0	192.1 ± 16.0	213.5 ± 16.0	250.5 ± 28.6	<.0001
Triglycerides (mg/dL)	82.7 (58.6–132.3)	91.0 (68.4–131.6)	102.3 (76.7–136.8)	119.5 (89.5–157.9)	<.0001
HDL‐C (mg/dL)	50.1 ± 13.4	51.3 ± 12.9	52.4 ± 11.9	53.7 ± 11.0	<.0001
Fasting blood glucose (mg/dL)	90.3 ± 9.9	91.9 ± 10.3	91.9 ± 10.1	93.9 ± 10.2	<.0001
2‐h post‐load glucose (mg/dL)	113.8 ± 30.6	117.7 ± 31.0	121.3 ± 30.1	126.8 ± 32.0	<.0001
Fasting serum insulin (μIU/mL)	5.80 (3.80–8.40)	6.40 (4.40–9.20)	6.80 (4.70–9.36)	7.00 (5.00–9.70)	<.0001
HOMA‐IR	1.27 (0.84–1.94)	1.44 (0.97–2.11)	1.51 (1.05–2.19)	1.61 (1.12–2.27)	<.0001
Hypertension, *n* (%)	642 (48.3)	711 (55.0)	768 (58.0)	819 (62.1)	<.0001
Dyslipidemia, *n* (%)	375 (28.2)	312 (24.1)	347 (26.2)	945 (71.4)	<.0001

*Note*: Data are presented in as means ± standard deviations (SDs) for normally distributed variables or as medians (interquartile ranges) for skewed variables. Categorical variables are presented as numbers and proportions.

Abbreviations: HDL‐C, high‐density lipoprotein cholesterol; HOMA‐IR, homeostatic model assessment for insulin resistance.

### 3.2 Baseline LDL‐C levels and incident diabetes

During a median follow‐up of 4.4 years, 652 participants (12.4%) developed diabetes. The cumulative proportion of incident diabetes increased significantly across LDL‐C quartiles. After adjustment for age and sex, there was a significant and positive association between serum LDL‐C levels and incidence of diabetes (model 1, odds ratio [OR] for quartiles 2 vs. 1: 1.42, 95% confidence interval [CI] 1.10–1.82; OR for quartiles 3 vs. 1: 1.50, 95% CI 1.17–1.92; OR for quartiles 4 vs. 1: 1.61, 95% CI 1.26–2.06; all *p* <.01) (Table 2). The results remained significant after additional adjustment for education level, current smoking, current drinking, physical activity, BMI, hypertension, triglycerides, and HDL‐C based on model 1. Similarly, the results were essentially unchanged after accounting HOMA‐IR (model 3).

**TABLE 2:** Risks of incident diabetes according to baseline levels of LDL‐C.

		Model 1		Model 2		Model 3	
	No. of cases/participants (%)	OR (95% CI)	*p*	OR (95% CI)	*p*	OR (95% CI)	*p*
Quartiles							
Q 1	121/1329 (9.1)	1.00 (reference)	–	1.00 (reference)	–	1.00 (reference)	–
Q 2	162/1295 (12.5)	1.42 (1.10–1.82)	.0065	1.32 (1.02–1.70)	.0348	1.30 (1.01–1.68)	.0453
Q 3	179/1326 (13.5)	1.50 (1.17–1.92)	.0014	1.35 (1.05–1.74)	.0201	1.33 (1.04–1.72)	.0261
Q 4	190/1324 (14.4)	1.61 (1.26–2.06)	.0001	1.32 (1.02–1.71)	.0320	1.30 (1.01–1.68)	.0431
*p* for trend	<0.0001	/	.0002	/	.0438	/	.0582
Continuous variable							
Per 1‐SD increment	652/5274 (12.4)	1.19 (1.10–1.29)	<.0001	1.13 (1.04–1.23)	.0045	1.12 (1.03–1.22)	.0110

*Note*: Model 1 was adjusted for age and sex. Model 2 was further adjusted for family history of diabetes, education level, current smoking, current drinking, physical activity, body mass index, hypertension, triglycerides, and HDL‐C, based on model 1. Model 3 was further adjusted for HOMA‐IR, based on model 2.

Abbreviations: CIs, confidence intervals; HDL‐C, high‐density lipoprotein cholesterol; HOMA‐IR, homeostatic model assessment for insulin resistance; ORs, odds ratios.

**FIGURE 2:**
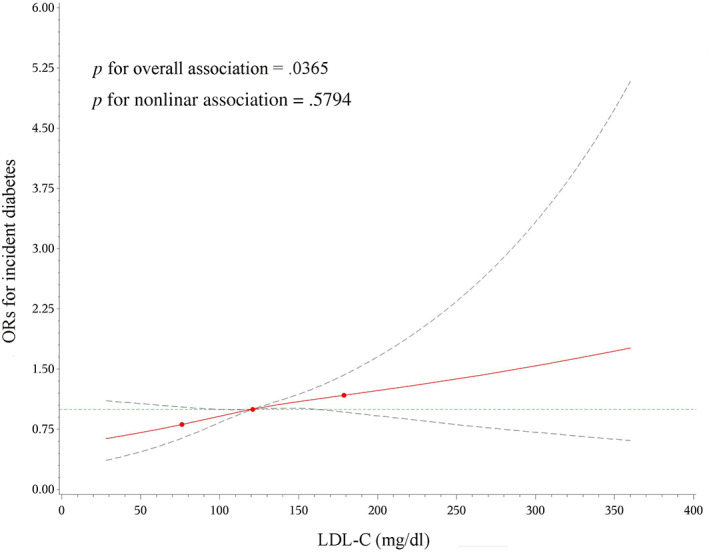
Restricted cubic spline of multivariable‐adjusted odds ratios for the association between baseline LDL‐C concentrations and incident diabetes. Adjusted for age, sex, family history of diabetes, education level, current smoking, current drinking, physical activity, body mass index, hypertension, triglycerides, HDL‐C, and HOMA‐IR. HDL‐C, high‐density lipoprotein cholesterol; HOMA‐IR, homeostatic model assessment for insulin resistance; LDL‐C, low‐density lipoprotein cholesterol; OR, odds ratio.

The cubic spline representing the shape of the association between serum LDL‐C concentrations and the incidence risk of diabetes demonstrated a nearly linear association (Figure 2). The risk of diabetes increased 12% in association with per 1‐SD increment in LDL‐C levels (OR 1.12, 95% CI 1.03–1.22, *p* =.01110) (Table 2).

### 3.3 Subgroup analysis

**FIGURE 3:**
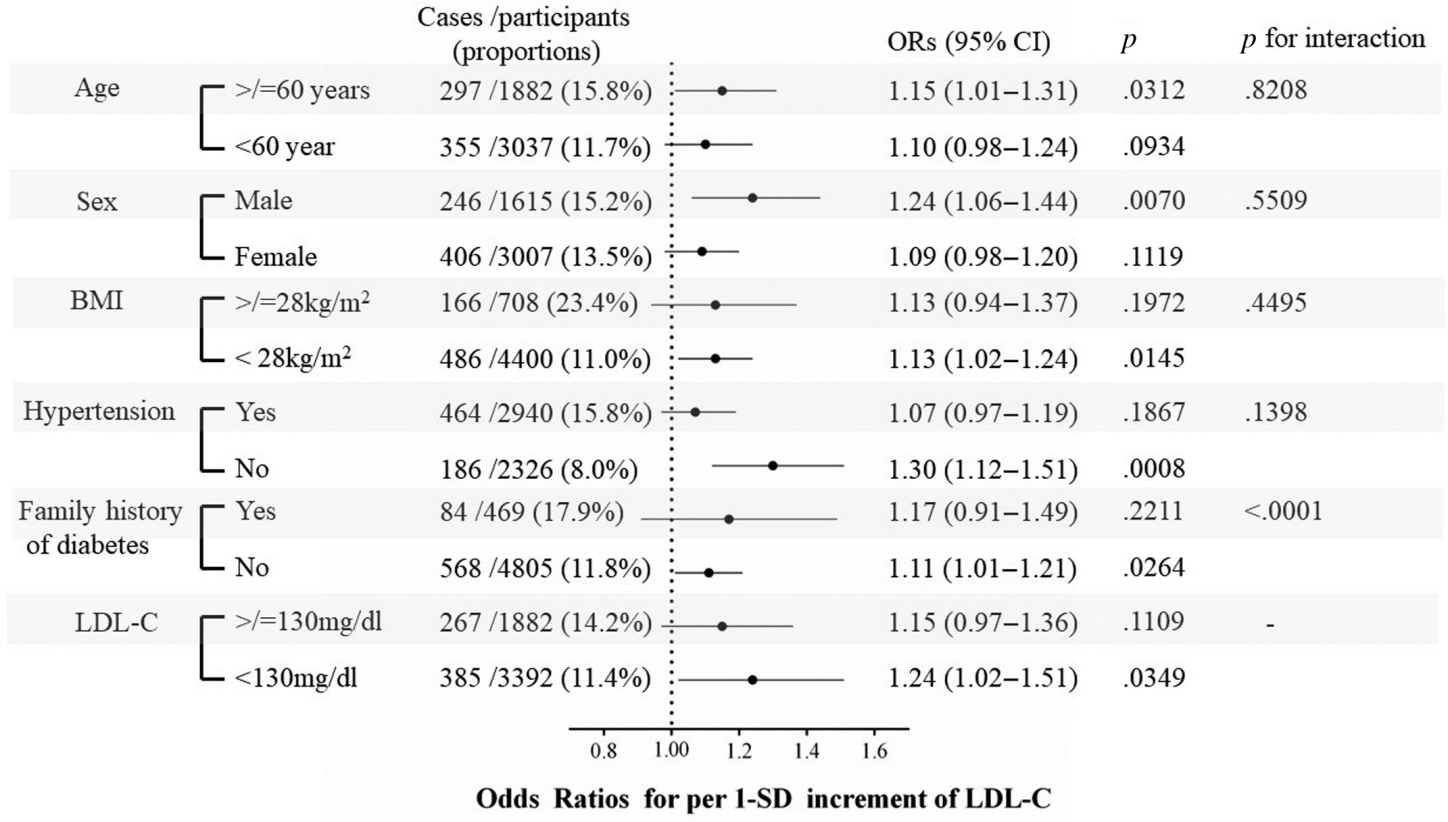
ORs and 95% CIs of incident diabetes in association with 1‐SD LDL‐C increase in subgroups. BMI, body mass index; CIs, confidence intervals; LDL‐C, low‐density lipoprotein cholesterol; ORs, odds ratios.

The association between LDL‐C levels and risks of incident diabetes was similar for subgroups of age, sex, BMI, baseline hypertension status, and LDL‐C levels (all *p* values for interaction >.05). However, LDL‐C levels showed a significantly stronger (*p* <.0001 for interaction) association with the incidence of diabetes in those without a family history of diabetes than in those with a family history of diabetes. Specifically, even in participants with normal LDL‐C levels (<130 mg/dL), a 1‐SD increase in LDL‐C levels was significantly associated with a higher risk of diabetes development (OR 1.24, 95% CI 1.02–1.51) (Figure 3).

### 3.4 Sensitivity analysis

We conducted several sensitivity analyses to assess the robustness of our findings. The risk of incident diabetes associated with 1‐SD increase in LDL‐C levels was 1.14 (1.04–1.24) after excluding participants who had a CVD history at baseline, 1.10 (1.00–1.21) after excluding participants who had impaired fasting glucose at baseline, 1.15 (1.01–1.32) after excluding participants who had impaired glucose tolerance at baseline, and 1.12 (1.03–1.23) after excluding participants who were using lipid‐lowering medications at follow‐up, due to the concern that statins might increase glucose levels. Therefore, findings from sensitivity analyses were similar to findings from the main analysis.

## 4 DISCUSSION

In this community‐based population study of 5274 Chinese adults aged ≥ 40 years, we observed that higher serum LDL‐C levels were associated with an elevated risk of developing new‐onset diabetes over an approximately 4.4 years of follow‐up, after adjustment for established risk factors including baseline HOMA‐IR. Sensitivity analyses by excluding participants with baseline CVD or prediabetes revealed similar findings.

Several cross‐sectional studies have examined the relationship between serum LDL‐C concentrations and the prevalence of diabetes. One study in a Chinese population showed a U‐shaped association, and in participants with LDL‐C concentrations < 130 mg/dL, lower LDL‐C levels were associated with a higher risk of diabetes.^7^ However, this study was limited by its cross‐sectional design and the inclusion of only patients with hypertension. Two previous studies have examined the prospective association between LDL‐C and incidence of diabetes, one in the American population and the other in Iranians. Our findings were consistent with those from the Isfahan diabetes prevention study (*n* = 1819), in which higher levels of LDL‐C were positively associated with the incidence of diabetes during a mean 10‐year follow‐up.^13^ In contrast, in the Framingham Heart Study (*n* = 14 120), baseline LDL‐C levels were inversely associated with the incidence of diabetes over a mean 4.5‐year follow‐up^5^. Potential reasons for these inconsistent findings include different genetic backgrounds and varying lifestyle factors related to LDL‐C, such as dietary habits. Moreover, different definitions were used to diagnose diabetes; for example, most studies did not measure PPG during an OGTT.^5^

The relationship between LDL‐C and diabetes has primarily been reported in clinical trials of statins,^3^ which showed that statin therapy was associated with an increased risk of developing diabetes. Regulation of cholesterol homeostasis in mammals at the individual level includes three aspects: de novo synthesis of endogenous cholesterol, absorption of exogenous cholesterol (such as bile acid and food), and conversion and efflux of cholesterol.^14^ Interactions were observed among the three pathways. For example, blocking a channel such as the NPC1L1 protein to lower intestinal cholesterol uptake might lead to higher endogenous cholesterol production.^15^ The phenotype of plasma glucose in cholesterol‐related‐gene knockout mice is also dependent on diet habits.^16^ Meanwhile, several important cholesterol metabolism‐related genes have been discovered, such as LIMA1, which plays a critical role in cholesterol absorption in a recent report.^17^ However, Mendelian randomization analysis using cholesterol metabolism‐related genes does not include LIMA1.^18^, ^19^ Therefore, different genetic instruments used to assess the relationship between LDL‐C levels and diabetes might possibly lead to different conclusions.

The main strength of the current study is its prospective design and a large sample size. Our study is the first prospective investigation of the association between baseline LDL‐C levels and new‐onset diabetes in the Chinese population. In addition, diabetes was defined by both fasting and 2‐h post‐load glucose levels at both baseline and follow‐up examinations. There are several limitations of this study. First, we did not distinguish between type 1 and type 2 diabetes. However, type 2 diabetes accounts for more than 90% of all diabetes in Chinese population.^20^ Second, although we adjusted for many confounding variables, residual confounders may still exist. Third, the follow‐up duration is relatively short. More incident cases of diabetes have to be accumulated for the analysis, especially at the lower end of LDL‐C concentrations. Finally, the generalizability is limited to Chinese community residents aged ≥ 40 years.

In conclusion, the current study found that higher LDL‐C levels are linearly associated with higher risks of diabetes during a median of 4.4‐years follow‐up in middle‐aged and elderly Chinese adults. Effective health management strategies should be implemented to prevent and control diabetes in patients with elevated LDL‐C levels.

## CONFLICT OF INTEREST STATEMENT

We declare that we have no conflicts of interest.

## Data Availability

All data used in the current study are available upon reasonable request addressed to the corresponding author.
